# Entwicklung der Nasennebenhöhlenchirurgie in Österreich und der Schweiz: Vergangenheit – Gegenwart – Zukunft

**DOI:** 10.1007/s00106-024-01539-3

**Published:** 2025-01-02

**Authors:** Erich Vyskocil, Axel Wolf, Dominik Hinder

**Affiliations:** 1 Abteilung für Hals‑, Nasen- und Ohrenkrankheiten, Medizinische Universität Wien, Währinger Gürtel 18–20, 1090 Wien, Österreich; 2 Praxis HNO Wolf Graz, Graz, Österreich; 3 Zentrum für HNO- und plastische Gesichtschirurgie, ORL-Zentrum Klinik Hirslanden, Zürich, Schweiz

**Keywords:** Nebenhöhlenchirurgie, Endoskopische Schädelbasischirurgie, Technologischer Fortschritt, Präoperative Planung, Chirurgisches Training, Endoscopic sinus surgery, Endoscopic skull base surgery, Technological advances, Preoperative planning, Surgical training

## Abstract

Unseren Vorgängern und dem technischen Fortschritt ist es zu verdanken, dass wir heute unseren Patienten eine sichere und moderne Nasennebenhöhlen(NNH)-Chirurgie anbieten können. Die vorliegende Arbeit gibt einen Überblick über die geschichtliche Entwicklung der NNH-Chirurgie in Österreich und der Schweiz und über den dynamischen Fortschritt dieser Disziplin im internationalen Kontext. Während die Indikation ursprünglich auf die Therapie von entzündlichen Erkrankungen begrenzt war, sind heute endoskopische Eingriffe im Bereich der vorderen Schädelbasis, der Orbita und auch von sinunasalen Tumorerkrankungen möglich, was zu einer geringeren Morbidität geführt hat. Das Konzept der funktionellen endoskopischen NNH-Chirurgie (FESS) mag einfach erscheinen, aber die Variabilität der Anatomie der NNH und das breite Spektrum der Krankheiten stellen eine Herausforderung dar. Daher sollte die NNH-Chirurgie keine Gelegenheitsoperation sein und nur von gut ausgebildeten Chirurgen durchgeführt werden, um Krankheitsrezidiven und ökonomischen Folgekosten vorzubeugen. Ein standardisiertes chirurgisches Training für angehende NNH- und Schädelbasischirurgen ist von entscheidender Bedeutung. Die präoperative Planung mittels systematischer Analyse der Computertomographie(CT)-Aufnahmen ist ein wesentlicher Faktor, um optimale Ergebnisse zu erzielen und intraoperative Komplikationen zu vermeiden. Das pathophysiologische Verständnis von sinunasalen Erkrankung und neue medikamentöse Therapien wie monoklonale Antikörper ermöglichen auch jener kleinen Subgruppe von Patienten, die von einer Kombination aus operativer Sanierung in Kombination mit medikamentöser Langzeittherapie nicht profitieren, exzellente Ergebnisse. Die dynamische Entwicklung der endoskopischen NNH-Chirurgie in den letzten Jahrzehnten zeigt das Potenzial dieses Bereichs für die kommenden Dekaden.

## Abstract (en)

Thanks to our predecessors and technical progress, we can nowadays offer our patients a safe and modern paranasal sinus surgery. This article provides an overview of the historical development of paranasal sinus surgery in Austria and Switzerland and to shed light on the dynamic progress of this discipline in an international context. Paranasal sinus surgery has undergone significant change in recent decades, driven by rapid technological advances that have significantly improved surgical procedures and operative outcomes. While indications were originally limited to treatment of inflammatory diseases, today, endoscopic procedures cover interventions in the area of the anterior skull base, the orbit and sinonasal tumors. The concept of functional endoscopic sinus surgery (FESS) may seem simple, but the anatomic variability, especially in the area of the frontal sinus, as well as the wide spectrum and severity of diseases can be a challenge. Therefore, paranasal sinus surgery should not be a casual operation and should only be performed by well-trained surgeons to prevent disease recurrence as well as medical and economic follow-up costs. Standardized surgical training for aspiring paranasal sinus and skull base surgeons is critical. Preoperative planning through systematic analysis of CT images is an essential factor to achieve optimal results and to avoid intraoperative complications. The pathophysiological understanding of sinonasal disease and the recent developments of new drug therapies such as monoclonal antibodies also enable excellent results in the small subgroup of patients who do not benefit from a combination of surgical rehabilitation and long-term drug therapy. The dynamic development of endoscopic paranasal sinus surgery in recent decades shows the potential of the field for the coming decades.

## Historischer Rückblick

Erste Beschreibungen zur Chirurgie der Nasennebenhöhlen (NNH) aus Österreich und der Schweiz finden sich anfangs des 20. Jahrhunderts. Charakteristisch für die damalige Zeit waren die paranasalen Zugänge zum Ethmoid und Sphenoid, welche erstmalig 1912 von Ottokar Chiari aus Wien beschrieben und eingeführt wurden. Beim paranasalen Zugang wurde der Hautschnitt ähnlich einer lateralen Rhinotomie angelegt, die Präparation direkt unter das Periost fortgesetzt, der Tränensack lateralisiert und nach Entfernung des Knochens zwischen Processus frontalis der Maxilla und der Orbita direkt ins Ethmoid eingegangen. Felix Nager, Gründer vom Otorhinolaryngologie(ORL)-Departement am Universitätsspital Zürich [1] berichtete 1919 über die von Chiari beschriebene Technik als Zugangsweg zur Hypophyse. Es finden sich erste Hinweise zum aktiven Wissensaustausch zwischen Chiari und Nager zu Beginn des 20. Jahrhunderts. Im Jahr 1940 berichtete Nager über Resultate von 39 Hypophyseneingriffen mit paranasalem Zugang, wobei nur einer der Patienten verstarb und die Todesursache als Herzversagen nach Pneumonie angegeben wurde. Aufgrund dessen beurteilte Nager die Operationstechnik von Chiari als sicher [2]. Hier bleibt zu erwähnen, dass die paranasalen NNH-Eingriffe üblicherweise mit der Stirnlampe und nur selten mit dem Mikroskop durchgeführt wurden. Bei blutreichen Verhältnissen war die Sicherheit schwierig zu gewährleisten, da die Ausleuchtung der tiefgelegenen Strukturen der NNH und der Schädelbasis eine Herausforderung darstellte. Entsprechend berichtete Harris Mosher aus Boston 1929 über seine Erfahrungen zur Ethmoidektomie: „Theoretically, the operation is easy. In practice, however, it has proved to be one of the easiest operations with which to kill a patient.“ [3]. Im Gegensatz zu Chiari und Nager propagierte Mosher keine paranasale, sondern eine endonasale Technik.

Mitte des 20. Jahrhunderts kamen die ersten Operationsmikroskope auf den Markt, was die Entwicklung der NNH-Chirurgie beeinflusste. So berichtete Hans Heermann aus Essen im Jahr 1958 über die Vorteile des Mikroskops gegenüber der Operation mit der Stirnlampe. Genannt werden die bis zu 40-fache Vergrößerung vom dreidimensionalen Bild, die Möglichkeit zur Farbfotographie sowie ein vereinfachtes Demonstrieren vom Operationssitus den Assistenten gegenüber [4]. Dank der zweihändigen Technik konnte der Chirurg auch bei blutreichem Operationsfeld die anatomischen Landmarken identifizieren und sicher operieren. Das Operationsmikroskop begann sich in Deutschland zu etablieren, und weitere Erfahrungsberichte zur Technik, wie derjenige von Helmut Masing aus Erlangen, verliehen dem Mikroskop zunehmende Beliebtheit [5].

Die ausreichende Beleuchtung der Nase und der NNH war eine zentrale Herausforderung in der Entwicklung der NNH-Chirurgie. Hopkins erzielte in den 1950er-Jahren grundlegende Verbesserungen in der Optik der Endoskopie. Dazu gehörten eine vom Instrument getrennte Lichtquelle (extrakorporale Kaltlichtquelle) sowie die Lichttransmission mittels Glasfaserkabel (Fa. Karl Storz sen., Tuttlingen, Deutschland, „Storz-Hopkins-Endoskope“). Dadurch wurde eine verbesserte Auflösung mit hohem Kontrast, ein großes Sichtfeld trotz des kleinen Durchmessers des Endoskops und eine perfekte Farbtreue erzielt [6].

In Österreich spielten die Leistungen von Walter Messerklinger und Heinz Stammberger aus Graz eine entscheidende Rolle. Walter Messerklinger gilt als der Erste, der aus dem transnasalen endoskopischen Blickwinkel die Anatomie und Physiologie der lateralen Nasenwand systematisch beschrieb [6]. Seine in den 1950er-Jahren begonnenen Studien zeigten den komplexen Zusammenhang zwischen Pathologien im Bereich der Drainagewege der NNH [7] und waren wegweisend für das spätere, komplexe Verständnis der Pathophysiologie im Bereich der lateralen Nasenwand [8]. Paradoxerweise verzögerte sich die Veröffentlichung einiger grundlegender Arbeiten von Messerklinger, da diese von Verlagen als rein akademische Studien ohne praktischen Nutzen für das Fachgebiet beurteilt wurden [9]. Dabei standen u. a. die ostiomeatale Einheit und der natürliche Transport von Sekret über das Flimmerepithel (mukoziliäre Clearance) im Fokus. Des weiteren beobachtete Messerklinger, dass die Beseitigung von primären vorderen Siebbeinerkrankungen durch einen umschriebenen endoskopischen chirurgischen Eingriff im Bereich der ostiomeatalen Einheit dazu führte, dass sich selbst massive entzündliche Schleimhautpathologien in den angrenzenden großen NNH innerhalb weniger Wochen erholten, ohne dass diese Bereiche direkt operiert werden mussten [6, 7, 9]. Somit war die Operationstechnik speziell auf Primärerkrankungen im Bereich des Siebbeins ausgerichtet. Damit wurde das Konzept der funktionellen endoskopischen Nasenebenhöhlenchirurgie (FESS) wesentlich durch Messerklinger und Stammberger beeinflusst.

In der Schweiz wurde das Endoskop zunächst im französischsprachigen Teil eingeführt. Georges Terrier aus La Chaux-de-Fonds veröffentliche 1976 seine Resultate zur Korrelation vom endoskopischen Bild und dem histopathologischen Befund von Biopsien, welche in endoskopischer Technik aus der Kieferhöhle gewonnen wurden [10]. Terrier wurde u. a. von Bauer inspiriert, einem Wiener HNO-Kollegen, welcher 1958 über seine Resultate der endoskopischen Kieferhöhlenbiopsie berichtete [11]. Terrier blieb stets ein Verfechter des Endoskops, propagierte seine Techniken in der französischen, deutschen und italienischen Schweiz und darf als Schweizer Pionier der endoskopischen Rhinologie gelten. Terriers Techniken wurde von Jean-Paul Friedrich weiterentwickelt, wobei die Erkenntnisse mehrheitlich in französischer Sprache publiziert wurden [12]. Im Jahr 1991 publizierte Terrier ein englischsprachiges Buch *Rhinosinusal endoscopy: diagnosis and surgery*, welches zum Standardwerk über endoskopische Techniken in der Schweiz wurde [13].

Gleichzeitig gab es wegweisende Entwicklungen aus Deutschland im Bereich der mikroskopischen und endoskopischen Techniken. Diese wurden teilweise kombiniert, sodass die Operation mehrheitlich mit dem Mikroskop durchgeführt und der Operationssitus mit dem Endoskop kontrolliert wurde. Als Beispiel für diese Methode kann das „Fulda-Konzept“ von Wolfgang Draf genannt werden, welches bis heute die Stirnhöhlenchirurgie geprägt hat [14]. Erwähnenswert sind auch die Beschreibungen von Malte Wigand, welcher bereits 1989 ein umfassendes Buch über endoskopische Techniken veröffentlichte [15]. In der deutschen Schweiz wurden Anfang der 1980er-Jahre die NNH-Operationen noch mehrheitlich mit dem Mikroskop durchgeführt. Der konsequente Wechsel zum Endoskop wurde in Zürich von Thomas Fenner initiiert, welcher die Techniken von Wigand lernte und seine Erfahrungen im Jahr 1984 veröffentlichte [16].

In Österreich wurde das Konzept der modernen funktionellen NNH-Chirurgie entscheidend von Heinz Stammberger geprägt. Endoskopische Diagnose- und Operationsverfahren, die von Stammberger und seinen Kollegen entwickelt und verfeinert wurden, verbesserten die Sichtbarkeit, Präzision und damit die Ergebnisse der Operationen erheblich. Sein Buch *Functional endoscopic sinus surgery* aus dem Jahr 1991 gilt als Standardwerk für die Technik und hat die Verbreitung der endoskopischen Nasenchirurgie weiter vorangetrieben [6]. Im Zentrum der Lehre von Stammberger in Zusammenhang mit funktionellen Eingriffen stand stets der Erhalt natürlicher, nicht pathologisch veränderter Strukturen der Nase (z. B. Nasenmuscheln), die Adaptation der Ausdehnung der Operation auf das individuelle Erkrankungsbild von Patienten sowie die intraoperative Orientierung an natürlichen anatomischen Strukturen („lamellar principles“) [6].

Simmen begann ausschließlich am Bildschirm zu operieren und verbesserte dadurch die Ergonomie

Ein ähnliches Konzept verfolgte Daniel Simmen in der Schweiz. Vor seiner Zeit wurden die Eingriffe mehrheitlich mit direktem Blick ins Endoskop vorgenommen. Simmen begann ausschließlich am Bildschirm zu operieren und konnte dadurch die Ergonomie des Chirurgen deutlich verbessern. Gleichzeitig begann er die Durchführung jährlicher Operationskurse, verfeinerte die endoskopischen Techniken und veröffentlichte gemeinsam mit Nick Jones das Buch *Manual of endoscopic sinus surgery and its extended applications* im Jahr 2005 [17]. Im gleichen Jahr wurde von Hans Rudolf Briner das Konzept der bimanuellen Technik vorgestellt [18]. Diese benötigt einen Assistenten, welcher dem Chirurgen das Endoskop hält. Dadurch wird ein beidhändiges Operieren ermöglicht. Briner konnte zeigen, dass dadurch die Operationszeit bei Patienten mit chronischer Rhinosinusitis verkürzt wurde. Die bimanuelle Technik hat sich heutzutage in der endoskopischen Schädelbasischirurgie als Standard etabliert, wobei sie in der NNH-Chirurgie nur in einzelnen Zentren durchgeführt wird [19].

## Paradigmenwechsel in der endoskopischen NNH-Chirurgie

Bei der chronischen Rhinosinusitis (CRS) handelt es sich um eine heterogene und entzündungsgetriebene Erkrankung, bei der die Kontrolle von Entzündungs- und Umweltfaktoren eine entscheidende Rolle spielt. Mit dieser Erkenntnis veränderten sich auch die chirurgischen Prinzipien. Während in der Chirurgie der CRS ohne Nasenpolypen (Nicht-Typ-2-Entzündung) weiterhin der „funktionelle“ Aspekt der endoskopischen NNH-Operation im Fokus steht, zielen moderne chirurgische Verfahren für die CRS mit Polypen (Typ-2-Entzündung) nicht nur darauf ab, die Ventilation wieder herzustellen, sondern vielmehr den pathophysiologischen Prozess aus Entzündung und Mukostase zu unterbrechen. Der inflammatorische Mukus wird maximal entfernt und damit auch die Konzentration von Entzündungsmediatoren, die diesen Prozess unterhalten. Das wesentliche Ziel dieser Art von Chirurgie ist zudem die Eröffnung der NNH, um sie einer entzündungshemmenden topischen Therapie zugänglich zu machen [20].

Diese Ansätze kombinieren Drainage mit der maximalen Entfernung chronisch alterierter Schleimhaut

Alternative Konzepte zu limitierten Eingriffen im Bereich des ostiomeatalen Einheit beinhalten erweiterte Operationen im NNH-System. Diese Ansätze kombinieren das Konzept der Drainage mit der Wiederherstellung der mukoziliären Clearance sowie einer maximalen Entfernung der chronisch alterierten Schleimhaut [21, 22]. Je nachdem welche Areale betroffen sind, handelt es sich hierbei um eine Frontoethmoidektomie in Kombination mit einer maxilläre Antrostomie und ggf. einer Sphenoidotomie. Dieser chirurgische Ansatz wird als Full-House-FESS [23], Pansinusoperation [24] oder gemäß EPOS (European Position Paper on Rhinosinusitis and Nasal Polyps Group) als „Full-FESS“ [25] bezeichnet.

**Abb. 1:**
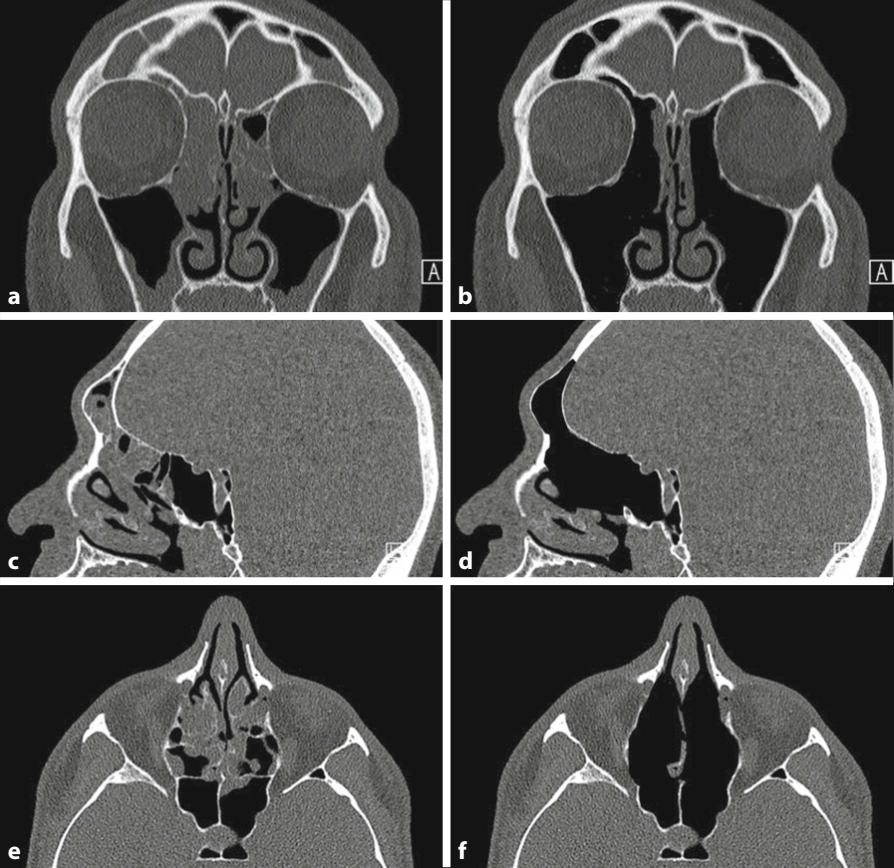
Konzept der Full-House-FESS (Fronto-Spheno-Ethmoidektomie). Präoperativer (**a**, **c**, **e**) vs. postoperativer Zustand (**b**, **d**, **f**). Koronarebene (**a**, **b**), Sagittalebene (**c**, **d**) und Transversalebene (**e**, **f**)

**Abb. 2:**
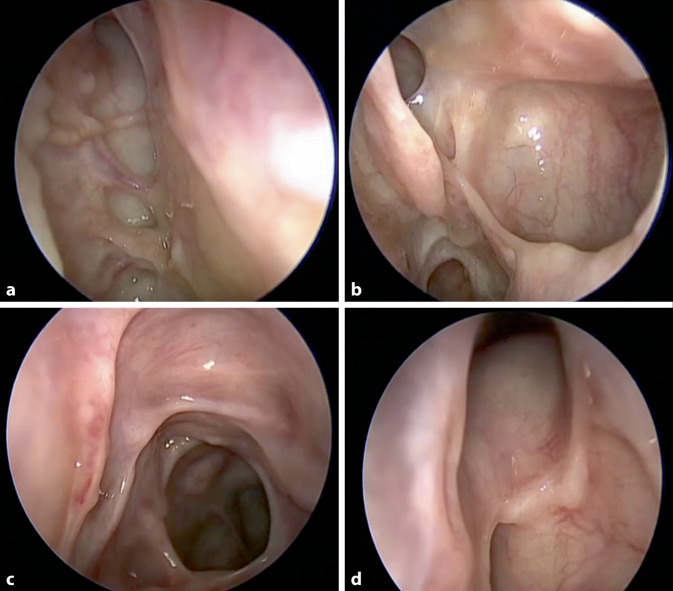
Postoperativer Befund nach Full-House-FESS (funktionelle endoskopische Nasennebenhöhlenchirurgie). Erläuterung s. Text. **a** Vorderes und hinteres Ethmoid mit einer in der Schädelbasis verlaufenden A. ethmoidalis anterior; **b** mittlere Muschel, Sinus maxillaris und *superior* die Antrostomie in den Sinus sphenoidalis, **c** Darstellung der Frontobasis mit großer Antrostomie in das Sphenoid, **d** Recessus und Sinus frontalis

Hierfür wird jegliche entzündlich veränderte Schleimhaut unterhalb der Schädelbasis und medial der Orbita entfernt (Abb. 1 und 2), im Sinne einer „kompletten“ vorderen und hinteren Ethmoidektomie, Kieferhöhlenfensterung, Sphenoidotomie und Eröffnung des Sinus frontalis. Den Autoren ist wichtig zu erwähnen, dass es sich beim vorgestellten Konzept nicht um eine „Reboot-Chirurgie“ [26] handelt. Vor allem im Bereich des Stirnhöhle muss ein maximaler anteroposteriorer Durchmesser erzielt werden, da hier am häufigsten Krankheitsrezidive auftreten [27]. Durch die Entfernung sämtlicher entzündlich veränderter Strukturen zwischen Orbita und Septum und unterhalb der Schädelbasis wird die Entzündungslast maximal reduziert und die Schleimhaut für topische Kortisonnasenduschen zugänglich gemacht (Abb. 1). In Abb. 2 zeigt sich eine komplett abgeheilte Nasenhaupthöhle mit weiten Osteotomien, die nach mehreren Monaten topischer Kortisontherapie eine vollständig abgeheilte Schleimhaut aufweist ohne Zeichen einer persistierenden Entzündung oder Repolyposis.

### Kombination mit medikamentöser Therapie

Ein wesentliches Ziel dieses Operationskonzeptes besteht darin, die maximal geöffneten NNH der topischen Therapie zugänglich zu machen. Dadurch wird es dem Patienten ermöglicht, eine Konversion von systemischen auf topische Steroide zu durchlaufen, wodurch potenzielle Langzeitnebenwirkungen von Steroiden minimiert werden [28]. Diese in Deutschland, der Schweiz und Österreich bisher weniger bekannte postoperative Routine sind sowohl hochvolumige als auch hochdosierte topische Kortisonspülungen. Dabei wird Kortison (Budesonid oder Mometason) in einer Nasenspülung appliziert und mehrere Wochen bis Monate einmal bis zweimal täglich verabreicht, um die postoperative Entzündung nach NNH-Eingriffen zu kontrollieren [29].

Ein wesentliches Ziel besteht darin, die geöffneten NNH der topischen Therapie zugänglich zu machen

Die Applikation von Kortisonnasensprays zeigen eine äußerst limitierte Fähigkeit zur Penetration der Nasennebenhöhle [30]. Die Kombination aus hochdosierten topischen Kortisonspülungen und erweiterter Chirurgie (Full-House-FESS, Full-FESS, Pansinusoperation) konnte die postoperativen Ergebnisse signifikant verbessern, wobei sich eine Reduktion der Rezidivraten bei Patienten mit einer chronischen Rhinosinusitis mit Typ-2- und Nicht-Typ-2-Entzündung in den Studien gezeigt hat [31–33].

**Abb. 3:**
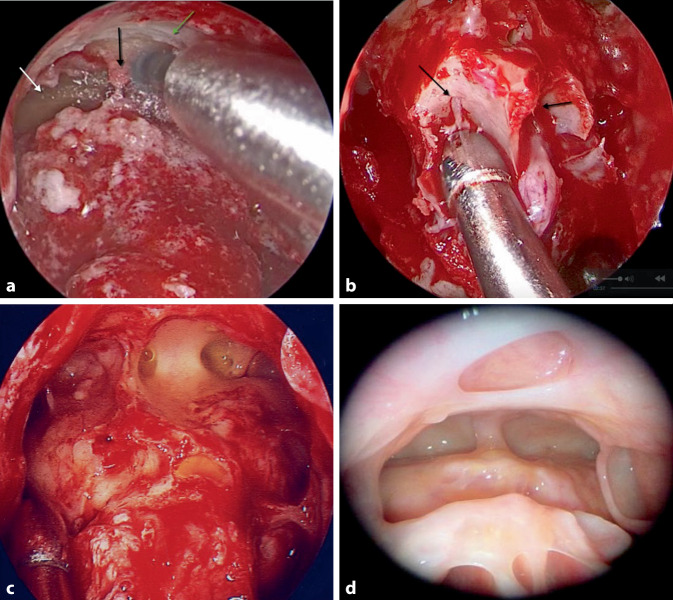
Frontal Drillout, Draf III. **a** Rechter Sinus frontalis (*weißer Pfeil*), Intersinusseptum (*schwarzer Pfeil*), knöcherne Sinus-frontalis-Vorderwand; **b** Darstellung des ersten olfaktorischen Neurons (*schwarzer Pfeil*) als posteriores Limit für die Entfernung von Knochen in Richtung Schädelbasis; **c** Operationssitus am Ende der Operation nach kompletter Entfernung des Sinus-frontalis-Bodens und maximaler Entfernung von Knochen im Bereich des „frontal beak“ mit der Haut als lateralem und posteriorem Limit der Dissektion; **d** postoperativer Zustand nach 6 Monaten

Besonders die schwierig zu therapierende Subgruppe der Patienten mit Asthma und Aspirinintoleranz scheint dabei von radikaleren Operationen, die über die Full-House-FESS hinausgehen, zu profitieren. So weist die Grad-6-Operation („endoscopic frontal sinus surgery“, EFSS) nach der neuen Klassifikation der Sinus-frontalis-Anatomie [34], auch bekannt als „Modified Lothrop“, Draf III oder „Frontal Drillout“ (Abb. 3) in Kombination mit einer Full-House-FESS verbesserte Ergebnisse bei Patienten mit AERD („aspirin-exacerbated respiratory disease“) auf und sorgt für eine hohe Erfolgsquote [35]. Die Rezidivrate sank von 90 % auf < 60 % nach der Primäroperation und die Revisionsrate von 89 auf 22,5 *% *[36]*.*

Es ist jedoch zu erwähnen, dass die Evaluation der optimalen Ausdehnung von Operationen im Zeitalter von Biologikatherapien in diese spezieller Patientengruppe weiterhin Gegenstand vieler laufender Studien ist.

### Fortschritte in der Bildgebung und Operationsplanung

**Abb. 4:**
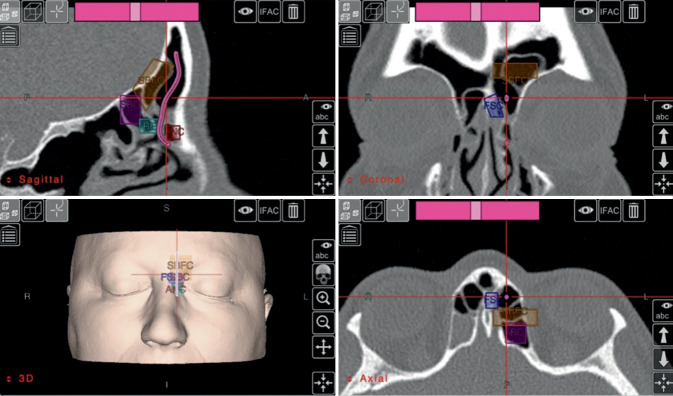
Building-Blocks-Konzept. Erläuterung s. Text

Von ganz entscheidender Bedeutung für das Verständnis sinunasaler Erkrankungen waren die Fortschritte in der Computertomographie(CT)-Bildgebung. Hochauflösende Bilder ermöglichen eine präzise Beurteilung der Anatomie, wodurch die Zellarchitektur genau analysiert und eine präoperative Planung ermöglicht wird. Mittels Bildgebungsprogrammen wie Osirix (Pixmeo, Bernex, Schweiz), Horos oder Building Blocks (Stryker ENT, Plymouth, MN, USA) (Abb. 4) können die CT-Daten in allen Schnittebenen betrachtet werden und ermöglichen es, ein dreidimensionales Verständnis der Anatomie zu erlangen. Jeder Operation sollte eine strukturierte Analyse der Anatomie vorausgehen, um so eine sichere und effektive Operation durchführen zu können.

Eine hilfreiche und systematische Option bei der präoperativen Analyse der CT ist das „CLOSE-System“

Eine hilfreiche und systematische Option bei der präoperativen Analyse der CT ist das „CLOSE-System“ [37], das die wesentlichen kritischen und potenziell gefährdeten Strukturen hervorhebt, die bei der Durchsicht der CT-Aufnahmen beurteilt werden sollten. Diese kritischen Strukturen können mithilfe der Gedächtnisstütze „CLOSE“ leicht erinnert und schnell abgerufen werden: *c*ribriforme Platte, *L*amina papyracea, *O*nodi-Zelle, *S*phenoid und (anteriore) *E*thmoidalarterie. Dieser Ansatz kann dazu beitragen, das Risiko chirurgischer Komplikationen zu verringern. Das in Abb. 4 dargestellte Building-Blocks-Konzept ermöglicht eine systematische Analyse der Anatomie des Sinus frontalis, auf deren Basis eine chirurgische Planung zur Entfernung der Zellen im Recessus und Sinus frontalis erstellt werden kann. Der natürliche Drainageweg und seine Relation zu den Zellen wird eingezeichnet und dargestellt, um intraoperativ entlang des natürlichen Drainagewegs eine sichere Entfernung der Zellen zu gewährleisten und kritische Strukturen wie die Schädelbasis oder Orbita nicht zu verletzen.

Intraoperativ ermöglicht die erwähnte Software einen Abgleich des Operationssitus mit der entsprechenden Anatomie in der CT. Das spielt eine entscheidende Rolle bei Stirnhöhleneingriffen, da dieser Sinus aufgrund seiner Lage und Nähe zu kritischen Strukturen wie Orbita und Schädelbasis der komplexeste Sinus ist. Mit der neuen Klassifikation der Zellen des Recessus und Sinus frontalis [34] (International Frontal Sinus Anatomy Classification, IFAC; Tab. 1) lassen sich die teilweise komplexen Zellformationen benennen und verstehen. Andererseits ermöglichen sie ein Verständnis, wie die Zellen den natürlichen Drainageweg der Stirnhöhle beeinflussen. Durch die Analyse der CT kann mental ein strukturierter Operationsplan entwickelt werden, der es ermöglicht die Instrumente entlang des Drainagewegs zu bewegen und so Schritt für Schritt die Zellen sicher und v. a. komplett zu entfernen. Zusätzlich zur Bildgebung haben moderne Navigationssysteme Einzug in die NNH-Chirurgie gehalten, die es ermöglichen, auf Basis der CT-Datensätze intraoperativ mit kalibrierten Sonden oder Instrumenten die Anatomie zu prüfen. Dieses Tool ist besonders bei komplexeren Eingriffen wie Frontal Drillouts/Draf III (Abb. 3) oder schädelbasischirurgischen Eingriffen von Bedeutung. Auch bei regulären Nebenhöhlenoperationen kann ihr Einsatz sinnvoll sein, ist aber natürlich kein Ersatz für das anatomische Wissen und das chirurgische Training des Operateurs.

**Tab. 1:** System der International Frontal Sinus Anatomy Classification zur Harmonisierung der Nomenklatur der Zellen [34]

Cell type	Cell name	Definition	Abbreviation
Anterior cells (push the drainage pathway of the frontal sinus medial, posterior or posteromedially)	Agger nasi cell	Cell that sits either anterior to the origin of the middle turbinate or sits directly above the most anterior insertion of the middle turbinate into the lateral nasal wall	ANC
	Supra agger cell	Anterior-lateral ethmoidal cell, located above the agger nasi cell (not pneumatizing into the frontal sinus)	SAC
	Supra agger frontal cell	Anterior-lateral ethmoidal cell that extends into the frontal sinus. A small SAFC will only extend into the floor of the frontal sinus, whereas a large SAFC may extend significantly into the frontal sinus and may even reach the roof of the frontal sinus	SAFC
Posterior cells (push the drainage pathway anteriorly)	Supra bulla cell	Cell above the bulla ethmoidalis that does not enter the frontal sinus	SBC
	Supra bulla frontal cell	Cell that originates in the supra-bulla region and pneumatizes along the skull base into the posterior region of the frontal sinus. The skull base forms the posterior wall of the cell	SBFC
	Supraorbital ethmoid cell	An anterior ethmoid cell that pneumatizes around, anterior to, or posterior to the anterior ethmoidal artery over the roof of the orbit. It often forms part of the posterior wall of an extensively pneumatized frontal sinus and may only be separated from the frontal sinus by a bony septation	SOEC
Medial cells (push the drainage pathway laterally)	Frontal septal cell	Medially based cell of the anterior ethmoid or the inferior frontal sinus, attached to or located in the interfrontal sinus septum, associated with the medial aspect of the frontal sinus outflow tract, pushing the drainage pathway laterally and frequently posteriorly	FSC

### Fortschritte im Bereich der Operationsinstrumente

Den Schlüssel für eine erfolgreiche Operationstechnik stellen moderne Instrumente dar – dies im Gegensatz zu reinen Greifinstrumenten, wie dem sog. Weil, der mitunter früher oft als einziges Instrument verwendet wurde. Während diese wenig Präzision ermöglichen, kann mit schneidenden Instrumenten die gesunde Schleimhaut erhalten und das Freilegen von Knochen verhindert werden, da dies zu vermehrten postoperativen Infektionen, stärkerer Verkrustung, länger dauernden Heilungszeiten und zu schlechteren Operationsergebnissen führen kann [31]*.* (Dies gilt v. a. im Recessus frontalis, der durch den engen Korridor äußerst anfällig für Stenosierungen nach Mukosastripping ist. Daher wurden feine gewinkelte Instrumente für Operation in diesem technisch anspruchsvollen Areal entwickelt, um eine komplette und schonende Chirurgie zu ermöglichen).

Ein Instrument, dass die NNH-Chirurgie deutlich optimierte, ist der Shaver oder Microdebrider

**Abb. 5:**
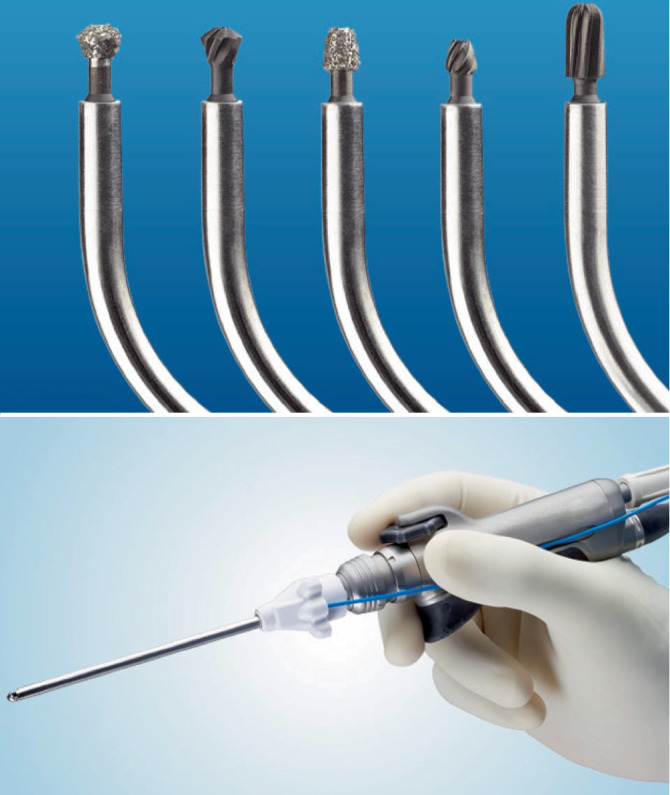
Microdebrider „Shaver“, der mit entsprechenden Aufsätzen auch als Bohrer verwendet werden kann. Mit freundl. Genehmigung © Fa. Medtronic GmbH, Meerbusch, Deutschland

Ein weiteres Instrument, dass die NNH-Chirurgie deutlich optimierte, ist der Shaver oder Microdebrider (Abb. 5). Dieser ist weltweit mittlerweile Standard und ermöglicht ein äußerst effizientes und präzises Operieren im Bereich der NNH. Dieses motorisierte Instrument wurde ursprünglich in der Orthopädie für Knorpelentfernung im Rahmen der Arthroskopie entwickelt und in den 1990er-Jahren für die NNH-Chirurgie adaptiert [38]. Moderne Microdebrider verfügen über gebogene Aufsätze mit rotierenden Köpfen, die auch im Bereich der Schädelbasis und des Recessus frontalis optimal eingesetzt werden können und eine atraumatische Entfernung von erkranktem Gewebe ermöglichen. Ihre Saug‑,Spül‑, Schneide- und Bohrfunktion, Letztere im Vorwärtsmodus, erlauben zahlreiche Verwendungsbereiche. Ihre Anwendung hat sich v. a. im angloamerikanischen Raum durchgesetzt, da hier im Rahmen von Fellowships seine sichere Anwendung gelehrt und weitergegeben wird. Trotz der hohen Effizienz bleibt zu erwähnen, dass der Microdebrider auch potenzielle Risiken wie intrakranielle oder intraorbitale Verletzungen mit sich bringt [39]. So genannte Saugspülbohrer ermöglichen zudem optimale Bedingungen für ausgedehntere chirurgische Verfahren im Bereich der Stirnhöhle (Draf und EFSS 4–6) oder der Schädelbasischirurgie.

Auch hochauflösende Kamerasysteme (4K) in Kombination mit endoskopischen Linsenspülsystemen und Navigationssystemen tragen dazu bei, dass die NNH-Chirurgie heute auf einem hohen technischen Niveau und sicher durchgeführt werden kann.

### Schädelbasis‑, Tumor,- und Orbitachirurgie

Die Indikationen der endoskopischen NNH-Chirurgie gehen heute weit über den Bereich der Behandlung der CRS hinaus. Seit den 1990er-Jahren gewann die endoskopische Hypophysenchirurgie an Popularität, sodass heute primär endoskopisch operiert wird, was zu einer verringerten Patientenmorbidität und zu kompletterer Tumorresektion führt [40]. Die endoskopischen Verfahren werden heute auch zur Resektion von sinunasalen Malignomen eingesetzt, die aufgrund von deutlich verbesserten Verschlusstechniken vergleichbare Überlebens- und Rezidivraten wie klassischere offene Ansätze haben [41]. Die Prinzipien der onkologischen Chirurgie lassen sich auf die endoskopischen Verfahren mit entsprechender Resektion und Sicherheitsabstand zur Tumorgrenze anwenden.

Auch im Bereich der Deckung von Schädelbasisdefekten und Liquorfisteln kam es seit Ende der 1980er-Jahre zu einem Paradigmenwechsel – weg von offenen Eingriffen hin zu geschlossenen Techniken –, als die ersten endoskopischen Schädelbasisverschlüsse veröffentlicht wurden. Während die ersten endoskopischen Serien mit nur kleinen Liquorfistel durchgeführt wurden, stehen heute dank verbesserter endoskopischer endonasaler Techniken wie dem nasoseptalen Lappen [42] eine Vielzahl von Verschlussoptionen zur Verfügung, um selbst große Rekonstruktionen erfolgreich durchzuführen. Während die endoskopische endonasale Rekonstruktion dieser großen Defekte im Jahr 2008 noch als Hochrisikoverfahren mit einer inakzeptablen postoperativen Liquorleckrate von bis zu 40 % [43] galt, liegt sie heute bei zirka 5 % [44].

Auch in der Tränenwegs- und Orbitachirurgie finden endoskopische Techniken ihre Anwendung

**Abb. 6:**
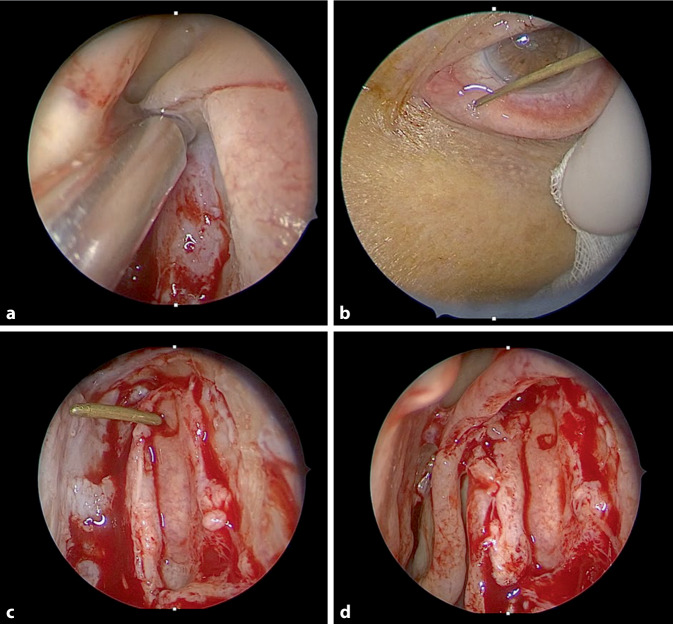
Endoskopische Dakryozystorhinostomie. **a** Entfernung des Processus maxillaris und maximale Exposition des Saccus nasolacrimalis; **b** Erweiterung des unteren Punctum nasolacrimale; **c** Marsupialisierung des Saccus nasolacrimale; **d** Adaptierung der sinunasalen Schleimhautränder mit dem marsupialisierten Saccus

Auch in der Tränenwegs- und Orbitachirurgie finden endoskopische Techniken ihre Anwendung. Während in den 1990er-Jahren die Erfolgsraten der endoskopischen Dakryozystorhinostomie (DCR; Abb. 6) bei enttäuschenden 83 % lagen, sind die Erfolgsraten mit der „powered endoscopic DCR“ auf eine Erfolgsquote von bis zu 97 % angestiegen [45]. Die Ergebnisse zeigen, dass sowohl die primäre DCR als auch die Revisionsoperation zuverlässige Langzeitergebnisse liefert, die den externen DCR-Verfahren überlegen sind. Der wesentliche Faktor ist eine komplette Marsupialisierung des vollständig freigelegten Ductus nasolacrimalis in Kombination mit einer Adaptierung der Tränenweg- und Nasenschleimhaut, die eine Heilung ohne Granulationsgewebe und nur minimer Narbenbildung ermöglicht.

Weitere Anwendungen der endoskopischen Orbitachirurgie sind die orbitale Dekompression bei der Graves-Orbitopathie, die Dekompression des Sehnervs und die Entfernung von Augenhöhlentumoren oder orbitaler kavernöser Hämangiome.

## Chirurgisches Training

Entscheidend für die Sicherheit der durchgeführten Operationen ist das genaue Verständnis der Anatomie der NNH und der vorderen Schädelbasis. Daher ist es von entscheidender Bedeutung, den jungen Generationen von NNH- und Schädelbasischirurginnen und -chirurgen eine fundierte theoretische, aber v. a. praktische Ausbildung anzubieten. Die Autoren sehen sich auch als Botschafter dieser Kultur der Wissensvermittlung in ihrem Gebiet und versuchen, im Rahmen von Kursen und Workshops die erworbenen Techniken zu vermitteln und weiterzugeben.

Durch den Fortschritt in 3‑D-Printverfahren ist es heute erstmals möglich, Gewebeimitationen auf sehr hohem Niveau herzustellen [46].

**Abb. 7:**
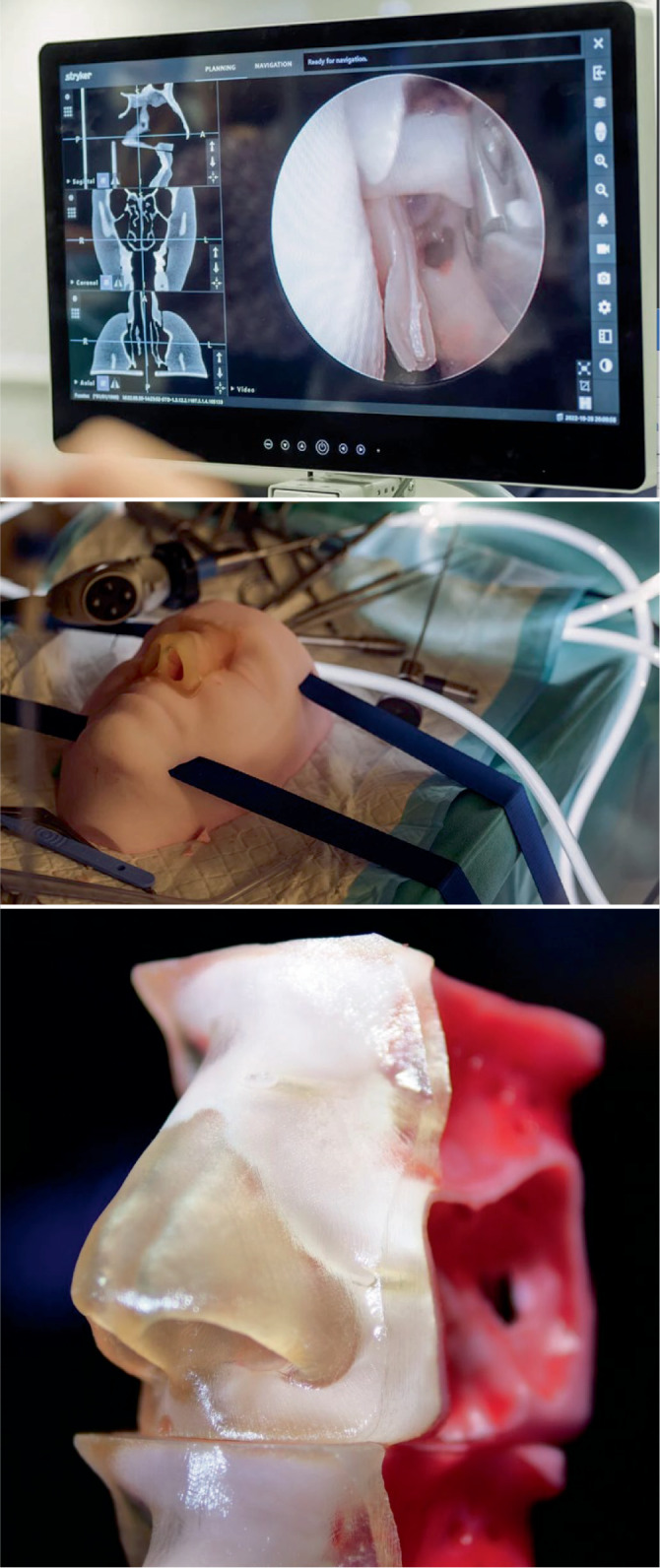
3‑D-gedruckte Nasennebenhöhlenmodelle mit standardisierter Anatomie auf Basis realer Patientendatensätze mit der Möglichkeit einer schrittweisen Annäherung von einfacher hin zu äußerst komplexer Anatomie des Sinus frontalis. (Mit freundl. Genehmigung © Fa. Fusetec, Adelaide, Australien)

Ein Vorteil von künstlichen Modellen ist, dass im Gegensatz zu Kadavern dieselbe standardisierte Anatomie vorliegt, die wiederum auf Basis realer Patientendatensätze erstellt wurde. Dies ermöglicht sowohl eine standardisierte Analyse der präoperativen CT-Untersuchungen, z. B. auf Basis des Building-Blocks-Systems [47], als auch eine Planung jedes einzelnen chirurgischen Schritts zur optimalen chirurgischen Bewältigung der vorliegenden anatomischen Voraussetzungen. Dies zu führt zu einer verbesserten Vorhersagbarkeit und Reproduzierbarkeit im Vergleich zu Kadavern [46]. Ein weiterer Vorteil einer standardisierten Anatomie im 3‑D-gedruckten Modell besteht darin, eine schrittweise Annäherung von einfacher hin zu äußerst komplexer Anatomie zu ermöglichen, was v. a. für die Stirnhöhle eine entscheidende Rolle spielt (Abb. 7).

Das Training an Kadavern kann die Haptik von biologischen Geweben vermitteln

Trotz dieser offensichtlichen Vorteile von Modellen hat das Training an Kadavern weiterhin einen großen Stellenwert, da es die Haptik von biologischen Geweben vermitteln kann. Wie erwähnt, sollte der Recessus frontalis komplett von obstruierenden Zellen und Septen befreit werden, da hier die höchste Rate von Polypenrezidiven auftritt [27]. Die jüngste Klassifikation der Sinus-frontalis-Anatomie ermöglichte eine Harmonisierung der Nomenklatur der Zellen im Recessus und Sinus frontalis. Die intuitive, einfache und anwenderfreundliche Verwendung der neuen Klassifikation in Kombination mit Bildgebungsprogrammen zur Analyse der Anatomie ermöglichen eine strukturierte und systematische Planung der Chirurgie im Sinus frontalis. Durch ein fundiertes Training sollte die zukünftige Generation von Kolleginnen und Kollegen den Patienten eine moderne und sichere NNH-Chirurgie anbieten können.

## Fazit für die Praxis

- Für angehende Nasennebenhöhlen(NNH)- und Schädelbasischirurgen ist ein standardisiertes chirurgisches Training von entscheidender Bedeutung.
- Um optimale Ergebnisse zu erzielen und intraoperative Komplikationen zu vermeiden, stellt die präoperative Planung mittels systematischer Analyse der Aufnahmen aus der Computertomographie (CT) einen wesentlichen Faktor dar.
- Neuere Entwicklungen bei medikamentösen Therapien, z. B. monoklonale Antikörper, ermöglichen auch gute Ergebnisse bei Patienten, bei denen es trotz operativer Sanierung und medikamentöser Langzeittherapie nicht zur Besserung gekommen ist.
- Die technischen und chirurgischen Entwicklungen in der endoskopischen NNH- und Schädelbasischirurgie stellen auch zukünftig ein grosses Potential dar.
- Das System der International Frontal Sinus Anatomy Classification (IFAC) harmonisiert die Nomenklatur der Zellen und ermöglicht eine intuitive und effiziente Analyse der Anatomie, auf deren Basis eine systematische präoperative Planung für Eingriffe im Sinus frontalis möglich wird.
